# Teaming up census and patient data to delineate fine-scale hospital service areas and identify geographic disparities in hospital accessibility

**DOI:** 10.1007/s10661-019-7413-4

**Published:** 2019-06-28

**Authors:** Peng Jia, Xinyu Shi, Imam M. Xierali

**Affiliations:** 1 Faculty of Geo-Information Science and Earth Observation (ITC), University of Twente, 7500 Enschede, The Netherlands; 2 International Initiative on Spatial Lifecourse Epidemiology (ISLE), 7500 Enschede, The Netherlands; 3 University College Twente, University of Twente, 7500 Enschede, The Netherlands; 4 Department of Educational Leadership and Policy, Graduate School of Education, University at Buffalo, The State University of New York, New York, NY 14260 USA; 5 Department of Family and Community Medicine, University of Texas Southwestern Medical Center, Dallas, TX 75390 USA; 6 Department of Geography and Anthropology, Louisiana State University, Baton Rouge, LA 70803 USA

**Keywords:** Accessibility, Census, Florida, GIS, HCUP, Hospital discharge, Hospital service area, Regionalization

## Abstract

The number of hospital beds per capita, an important measure of equity in healthcare availability and resource allocation, was found to vary across geographic areas in many countries, including the USA. The hospital service areas (HSAs) have proven to be more meaningful spatial units for studying health-seeking behaviors and health resource allocation and service utilization. However, when evaluating the geographical balance in ratios of hospital beds to population (HBtP), no existing HSA delineation methods directly consider the underlying population distribution. Using Geographic Information Systems (GIS), this study incorporated the State Inpatient Database with census data to develop a population-based HSA delineation method. The *census-derived HSAs* were produced for Florida and were validated by aggregating and comparing with the traditional flow-based HSAs. The difference in current ratios of HBtP between the most over- and under-served HSAs was approximately 60 times. Significant clusters of high and low ratios were found in Miami and Jacksonville metropolitan areas, respectively. Such results may be of interest to relevant stakeholders and contribute to planning and optimization of hospital resource allocation and healthcare policy-making. Furthermore, the discovery of a strong correlation between the numbers of hospital discharges and the population at ZIP code level holds a remarkable potential for affordable population estimation, especially in non-census years.

**Electronic supplementary material:**

The online version of this article (10.1007/s10661-019-7413-4) contains supplementary material, which is available to authorized users.

## Introduction

The number of hospital beds per capita is an important measure of healthcare resource allocation and equity in healthcare availability and accessibility (Anderson et al. 2005; Jia et al. 2015, 2017a; Krueger et al. 2009; Mansfield et al. 1999). Insufficient hospital beds cannot meet the demands of patients in local areas. Wherever healthcare resources are scarce, patients either incur long waits to be seen in local hospitals or travel to distant ones for care (Jackson et al. 2002). Under these circumstances, patients may be prevented from receiving timely care, and the patient–healthcare provider relationships can deteriorate (Hogan 1988; Jones et al. 2008; Moist et al. 2008; Wilbush 1974). Hospital beds per capita have been found to vary across geographic areas in many countries, including the USA, where the number of hospital beds per 1000 population was reported to be 2.9 on average in 2011, according to the World Bank collection of development indicators (Horev et al. 2004; Rodwin and Sandier 1993; Smith 1998; The World Bank 2017).

To mitigate the risk of negative impacts from scarce healthcare resources and optimize the current hospital resource allocation, the HBtP ratios need to be balanced across regions; this will also lead to adequate hospital capacity planning and accurate assessment of real-world healthcare utilization (Ashton et al. 1999; Fisher et al. 1994; Wennberg 1999). A basic solution is regionalization (Claval 1987), which divides an area (e.g., county, state/province, or country) into mutually exclusive spatial units with proper sizes and, based on this, evaluates the per capita share of healthcare resources. Nevertheless, it is argued that existing administrative boundaries are not aligned with the underlying travel behaviors of patients for healthcare purposes and therefore lack the functional healthcare applications presented by hospital service areas (HSAs) (Ashton et al. 1999; Jia et al. 2017a; Klauss et al. 2005; Zhang et al. 2012). An HSA unit is a relatively self-contained healthcare service area within which most of local hospitalization occurs (Center for Evaluative Clinical Sciences 1999). An overarching goal of defining HSAs is to create equitable care across these meaningful units (Jia et al. 2015), ultimately identifying and eliminating inequalities in hospital resource allocation and other factors associated with distant hospitalization (Jia and Xierali 2015).

Despite the availability of spatially finer-scale and socio-demographically homogeneous census units, e.g., census tract (CT), block group (BG), and block, HSAs resulting from previous delineation approaches are mainly aggregated by ZIP codes which are coarser than census units (Center for Evaluative Clinical Sciences 1999; Jia et al. 2015; Klauss et al. 2005). Such compromise is reasonable and accepted in most cases; after all, ZIP code is the finest geographic unit at which level patients’ locations of residence are reported in publicly available data (Agency for Healthcare Research and Quality 2011; Jia et al. 2017a). However, previous research suggests that substituting CTs for ZIP codes could significantly increase the accuracy of estimating future demand (Miller 1994). Furthermore, the importance of the population information within functional units has been stated for the success of the task of calculating hospital capacity (Patel et al. 2008). Lack of consideration of the population information in HSA delineation methodology may lead to too small or too large population within HSAs and, therefore, a biased relationship between supply and demand (Shortt et al. 2005). This study filled this gap by aggregating the spatial units reflecting underlying population distribution into HSAs, which would remarkably improve the usefulness of the derived HSAs, especially for such purposes as assessing per capita share of resources.

Against this background, this pilot study aimed to (1) integrate census data with hospital patient data to develop an approach for producing a set of population-based HSAs and (2) demonstrate the spatial disparities in current ratios of HBtP at the HSA level. This easy-to-implement approach bridges geographic and healthcare fields, which particularly serves healthcare planners for scrutinizing geographic variation in per capita share of healthcare resources on a finer geographic scale and for producing regionally and internationally comparable findings relative to the extent of equity in the distribution of healthcare resources.

## Methods

### Study area and data

Florida, consisting of 67 counties, is situated in the southeastern USA with three facets bordered by water: the Gulf of Mexico to the west, the Florida Straits between the USA and Cuba to the south, and the North Atlantic Ocean to the east. Therefore, compared to other individual states of the USA, the edge effect in terms of the tendency of patients to travel across state boundaries for hospital care is assumed to be minimized, which makes Florida an ideal study area for investigating patients’ healthcare travel patterns in one state. According to the 2010 Census (US Census Bureau 2014), a total population of roughly 18.8 million was registered in Florida, with a median age of 40.7 years and the percentages of age groups < 18, 18–44, 45–64, and ≥ 65 years being 21.3%, 34.4%, 27.0%, and 17.3%, respectively. The reference day used for the census was April 1, 2010 (National Census Day).

The State Inpatient Database (SID) has been assembled, edited, and standardized by the Agency for Healthcare Research and Quality (AHRQ) as part of the Healthcare Cost and Utilization Project (HCUP) (Agency for Healthcare Research and Quality 2011). Developed through a federal–state–industry partnership, the AHRQ disseminated the HCUP data to provide a large-scale resource of national, state, and all-payer healthcare data and to enhance nationwide comparability among independent health outcomes in different states. This study was based on a set of 2,376,743 inpatient discharge records in 2011 from all 221 general hospitals in Florida, as reported in the 2011 Florida SID data. The 2010 Florida ZIP code boundaries were used to match patients’ locations of residence in SID data. Thus, individual records were aggregated into the volume of discharges from each hospital to each ZIP code or, conversely, considered as patient-to-hospital travel flows.

The 2010 Census with numbers of total population within three-tier hierarchical census units in Florida includes 4245 CTs, 11,442 BGs, and 484,481 blocks. Previous studies have aggregated 983 ZIP codes into HSAs in Florida (Center for Evaluative Clinical Sciences 1999; Jia et al. 2017a, b). The BGs with the numbers of total population were chosen as the finest spatial units in this pilot study and considered as a good balance of being fine-scaled (relative to CTs) and not being overly and unnecessarily fine-scaled (disaggregating patients to blocks would introduce more uncertainties due to unknown locations of residence of patients).

The number of hospital beds in 2011 was derived from the 2013 American Hospital Association’s (AHA) survey files, ranging from 15 to 2170 with a mean of 252 and a median of 195. The primary and secondary road networks were available in Geographic Information Systems (GIS) format, published in 2014 by the Florida Department of Transportation. Prior to network analysis, preprocessing was conducted in the *ArcGIS* editing environment (Version 10.4.1, ESRI, Redlands, CA) to ensure a fully connected road network. As a baseline for comparison, the *Dartmouth-derived HSAs* in Florida, produced by a traditional flow-based method (Appendix 1), were obtained from Jia et al. (2015).

### Matching census and patient data

To assign each BG to the hospital discharging most patients in that BG (i.e., aggregating BGs into HSAs), the numbers of discharges within BGs are needed to be estimated. This necessitated building a relationship between the numbers of discharges and the population. The locational information of the patients was only available at ZIP code level. However, the population numbers within ZIP codes were not directly available from the census data and BG boundaries were not aligned with ZIP code boundaries (BGs were nested within CTs instead of ZIP codes). Therefore, an *areal weighting interpolator* (AWI) method (Goodchild and Lam 1980) was used to interpolate the population within ZIP codes from populated BGs. Implementation began with intersecting the ZIP code layer and the BG layer. Some BGs were completely located within ZIP codes, but some were divided into two or more intersected zones by ZIP code boundaries. The AWI method assumes that population is uniformly distributed within a BG. Thus, the population in each BG, if divided, was apportioned to each intersected zone on a basis of the areal proportion of that intersected zone over the BG. The estimated population in all intersected zones and BGs completely located within each ZIP code was then added to yield the total population in that ZIP code. As a result, a high correlation (Pearson’s *r* = 0.92) was found between the numbers of hospital discharges and the population at ZIP code level. Hence, a reasonable assumption was made that the percentage of discharges in a BG over a ZIP code approximated to the percentage of the population in that BG over that ZIP code.

For the sake of simplicity, BGs and the intersected zones by BGs and ZIP codes were both referred to as BGs without differentiation for the remainder of this study.

### Delineating census-derived HSAs

Before assigning each BG to the hospital discharging most of its patients, it is worth recalling that the traditional approaches of HSA delineation all assign a ZIP code to the hospital that either actually (Center for Evaluative Clinical Sciences 1999; Klauss et al. 2005) or potentially discharges the largest number of patients in that ZIP code (Jia et al. 2017b). An important motivation to replace ZIP codes with BGs as basic units, in addition to availability of the population numbers at BG level, is to better handle some uncertain cases where comparable percentages (e.g., 51% and 49%) of discharges in a ZIP code were from different hospitals at completely different locations, but that ZIP code was only assigned to one hospital (i.e., one that discharged 51% of patients). With that been said, those ZIP codes with a relatively large percentage of discharges from one hospital can still be reasonably assigned to that hospital without needing to be disassembled into BGs. Therefore, the percentage of discharges from each hospital to each ZIP code was computed based on all patient-to-hospital travel flows, and each ZIP code with the highest percentage of discharges (from hospital A) equal to or greater than two times the second highest percentage of discharges (from hospital B) was assigned to hospital A. Each continuous cluster of ZIP codes assigned to the same hospital, with that hospital located within the cluster, formed an initial HSA.

**Fig. 1:**
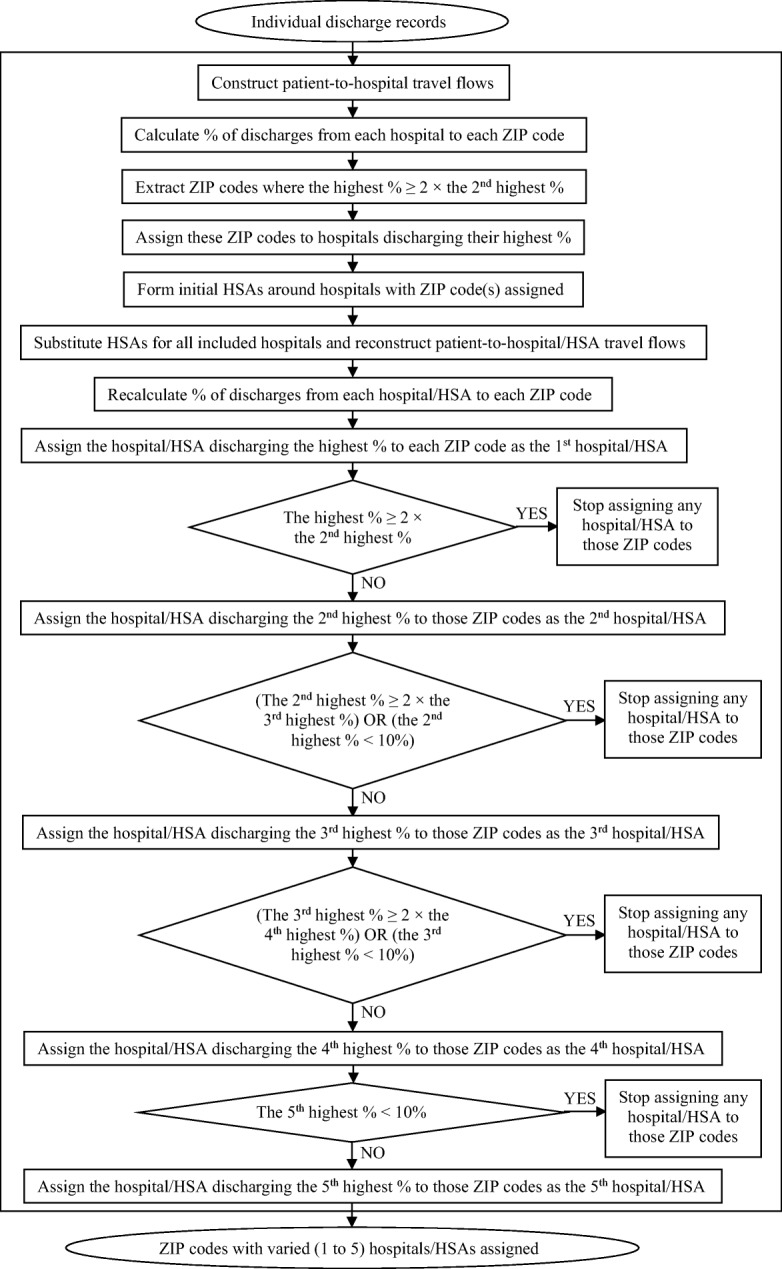
A flowchart of assigning hospitals to ZIP codes

Next, with each initial HSA substituting for the hospital(s) situated within it, patient-to-hospital flows were re-constructed and the percentage of discharges from each hospital/HSA to each unassigned ZIP code was re-computed. The hospital/HSA discharging the highest percentage of patients to a given ZIP code was assigned as the first hospital/HSA to that ZIP code. If that percentage was smaller than two times the next highest percentage of discharges from a different hospital/HSA, then this hospital/HSA was assigned as the second hospital/HSA to that ZIP code; otherwise, if that percentage was equal to or greater than two times the next highest percentage, or the next highest percentage was below 10%, then assigning ended. This procedure was repeated for each ZIP code until either assigning ended or the fifth hospital was assigned (Fig. 1). As a result, each ZIP code was assigned a varied number of hospitals/HSAs between one and five, with corresponding percentage(s) of discharges all equal to or greater than 10%.

To assign the unassigned ZIP codes which had been split up into BGs (and intersected zones) after the intersection process to their first–fifth hospitals/HSAs, two assumptions were made due to lack of the information on patients’ hospital selection: (1) patients tended to go to the nearest hospitals and (2) if there was a certain percentage of discharges from a hospital within a given ZIP code, those patients should most likely live in the nearest BGs (to the hospital) which contained the same percentage of the population over that ZIP code. The distance was measured along the shortest path on road networks from the population-weighted centroids of BGs (Appendix 2) to each hospital or to the population-weighted centroid of each HSA (Appendix 3). The BGs in each ZIP code were ranked by distance to each hospital/HSA.

**Fig. 2:**
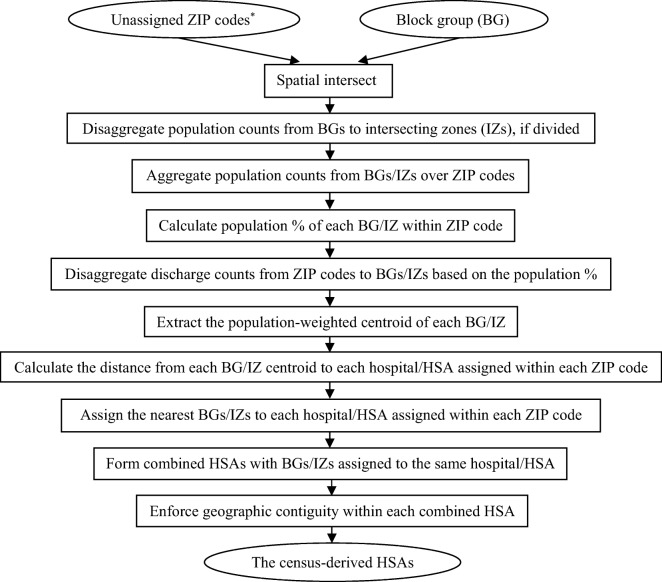
A flowchart of generating the census-derived hospital service areas (HSAs). Unassigned ZIP codes are the output of Fig. 1, i.e., those ZIP codes which were not assigned to hospitals as a whole, with varied (one to five) hospitals/HSAs assigned (asterisk)

In each ZIP code, starting from the first hospital/HSA, each hospital (i.e., BG in which the hospital was located) or HSA continued to merge with the nearest BG until the summed population proportion of the merged BGs reached the percentage of discharges from that hospital/HSA. As the sum of five (or less) highest percentages of discharges may be less than 100% within a ZIP code (i.e., some discharges were from other hospitals than these numbered hospitals/HSAs), the unassigned BGs in each ZIP code were allocated by default to its first hospital/HSA. Each cluster of BGs assigned to the same hospital/HSA formed a combined HSA. A visual examination was undertaken to enforce the geographic contiguity of all ZIP codes and BGs within each combined HSA (discontinuity by natural water bodies was allowed). The final output was named the *census-derived HSAs* (Fig. 2).

### Ratio of HBtP

The population in all ZIP codes and BGs within each HSA was summed to yield the population in that HSA. Then, the HBtP ratio was calculated for each HSA by dividing the total number of hospital beds by the number of the population within that HSA. *Local Moran’s I* (Anselin et al. 2006) was used to identify spatial clusters of HSAs with ratios similar in magnitude (i.e., clusters of high or low ratios) and spatial outliers (i.e., high ratios surrounded by low ratios, or conversely, low ratios surrounded by high ratios) across Florida, based on HSA locations and ratios simultaneously. Any adjacent spatial unit could have ecological, social, and health impacts on the target unit; hence, the first order rook contiguity was applied, which means that all HSAs sharing at least a point-length border with the target HSA are defined as neighbors (Jia et al. 2016). Additionally, the HBtP ratios were also calculated within counties with the clustering patterns examined based on the same contiguity rule.

### Comparison between HSAs

The HSA products are usually validated by measuring the degree of self-containment of HSAs, i.e., what percentage of patients within an HSA actually visit local hospital(s) within that HSA. For such purpose, a *localization index* (LI) is defined as the fraction of discharges of residents that occur within an HSA over all discharges living in that HSA (Klauss et al. 2005). To compare the census-derived HSAs with other HSA products, as done in previous studies (Jia et al. 2015; Klauss et al. 2005), a rule of LI ≥ 0.5 was applied to forcing each HSA with LI < 0.5 to be merged into an adjacent HSA, which either geographically encircled that HSA or discharged the second highest percentage of patients to that HSA. Merging and testing were iteratively conducted until the LIs of all HSAs ≥ 0.5.

A *t* test was used to assess whether there were significant differences between LIs of the aggregated census-derived HSAs and the Dartmouth-derived HSAs. A natural log transformation was conducted to alleviate the skewed distribution of two groups of LIs.

All statistical analyses were performed using SPSS version 24.0 (IBM Corp., Armonk, NY). Implementation of all spatial analyses was conducted in *ArcGIS* (Version 10.4.1, ESRI, Redlands, CA).

## Results

### Census-derived HSAs

**Fig. 3:**
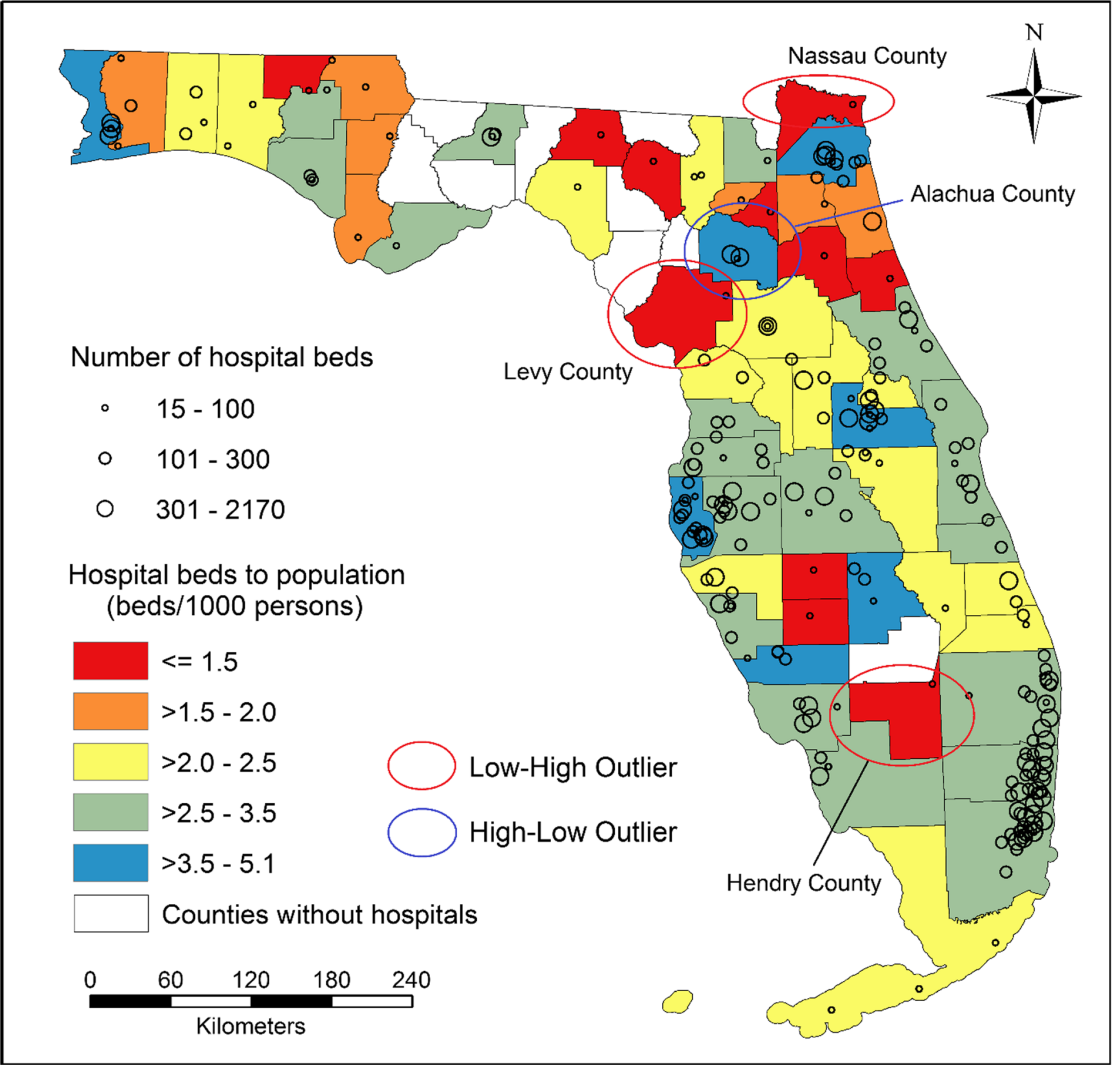
The ratios of hospital beds to population within counties and the distribution of hospitals with the numbers of hospital beds represented by the size of circles

The 221 general hospitals were located in 58 out of 67 counties in Florida, where the HBtP ratios (beds per 1000 people) ranged from 0.4 (Suwannee County) to 5.1 (Alachua County) (Fig. 3). Three counties (Hendry, Levy, and Nassau counties) with low ratios of HBtP were found to be surrounded by counties with significantly higher ratios, while Alachua County had a high ratio and was surrounded by counties with significantly lower ratios. There were 217,243 (983 × 221) patient-to-hospital flows naturally formed from 983 ZIP codes to 221 hospitals in Florida, of which 37,216 flows contained at least one hospital visit, according to the actual discharge records in the year 2011. Nearly half the ZIP codes (47.3%, 465/983) had the percentage of discharges from the first hospital equal to or greater than two times the percentage of discharges from the second hospital, which thus were assigned to their first hospitals. The unassigned 518 ZIP codes were divided into BGs and assigned to different initial HSAs.

**Fig. 4:**
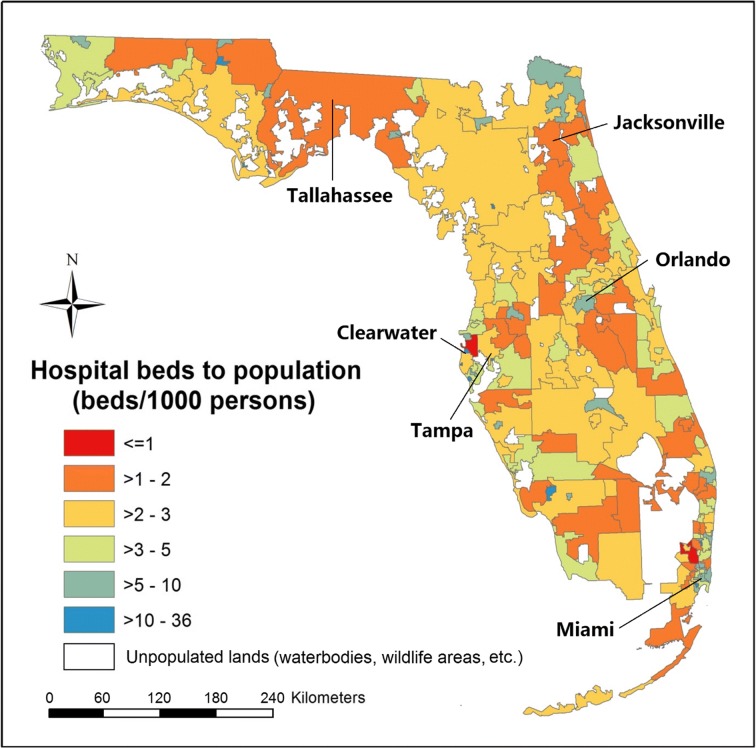
The ratios of hospital beds to population within the census-derived HSAs

**Fig. 5:**
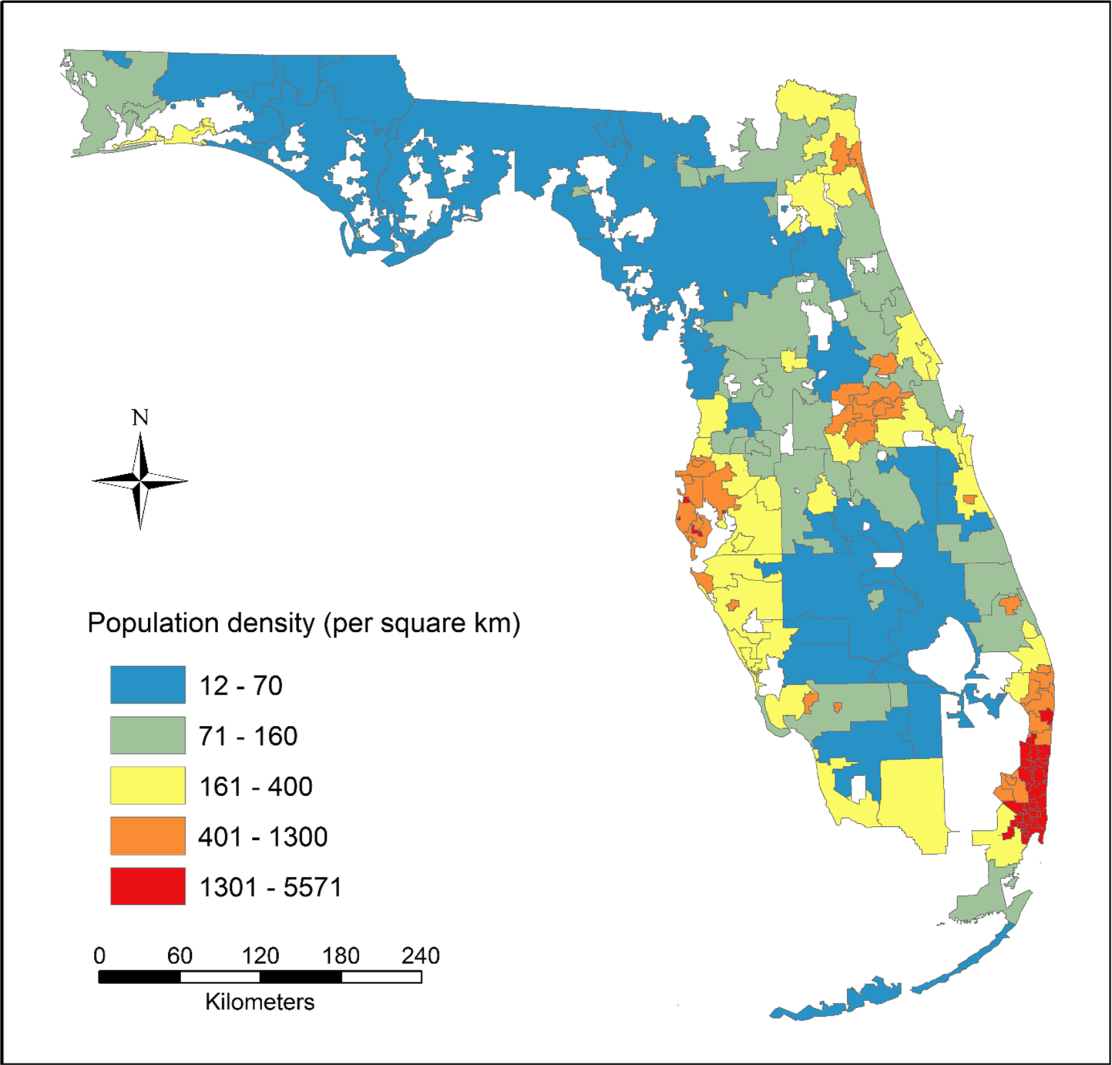
Population density within the census-derived HSAs

A total of 166 units were produced in the census-derived HSAs, with 80% of units including only one hospital, 13% including two, and 7% including three or more. Compared to the HBtP ratios at the county level, larger spatial heterogeneities in the ratios were observed across HSAs (Fig. 4), where the difference between the most over- and under-served areas was found to be approximately 60 times, i.e., 36.3 in Largo (to the south of Clearwater) versus 0.6 in Safety Harbor (to the east of Clearwater). Seventy-eight percent (130/166) of the HSAs showed ratios between one and five. A weak correlation (Pearson’s *r* = 0.24) was found between the HBtP ratios and the population density at HSA level (Fig. 5). The smaller-sized HSAs in metropolitan areas tended to have higher ratios. For example, a small significant cluster of high ratios was detected in Miami metropolitan area, while a cluster of low ratios was found in Jacksonville metropolitan area.

### Aggregation of census-derived HSAs

**Fig. 6:**
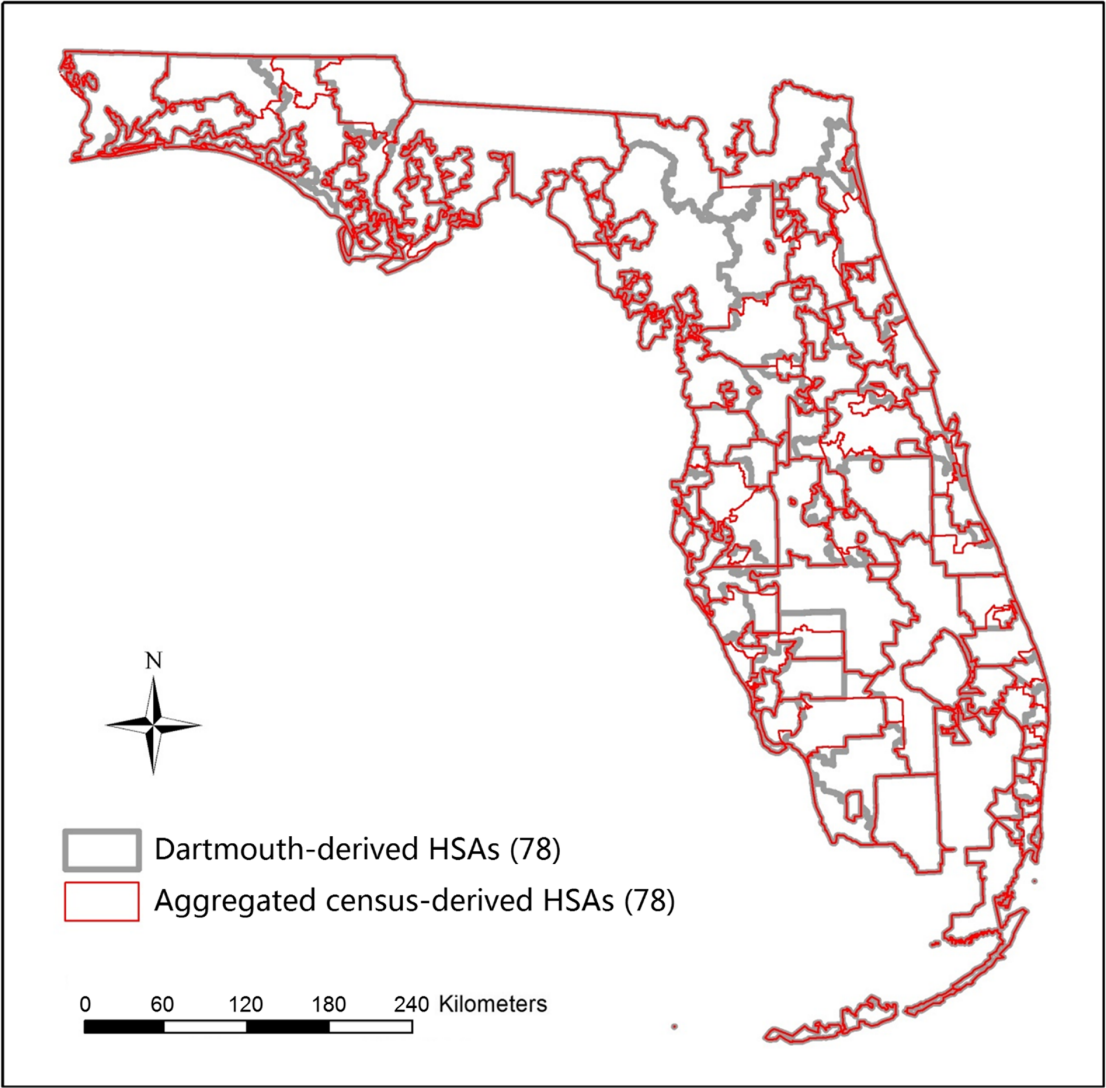
Boundaries of the Dartmouth-derived HSAs and aggregated census-derived HSAs

The 166 units in census-derived HSAs were combined into 78 units in aggregated census-derived HSAs after enforcing LI ≥ 0.5, which happened to be the same number of units in the Dartmouth-derived HSAs (Fig. 6). The boundaries of the aggregated census-derived HSAs were not aligned with the boundaries of the Dartmouth-derived HSAs, as boundaries of their underlying spatial units (BGs and ZIP codes) did not overlap. The LIs of the aggregated census-derived HSAs had a comparable mean (0.66 versus 0.65) and range (0.5 to 0.97 versus 0.5 to 0.93) with the LIs of the Dartmouth-derived HSAs. There were no differences in the log-transformed LIs for the aggregated census-derived HSAs (*M* = −0.431, SD = 0.182) and the Dartmouth-derived HSAs (*M* = −0.446, SD = 0.177); *t*(154) = −0.525, *p* = 0.600 (two-tailed). Thus, we considered that two sets of HSAs had no significant differences in the degree of self-containment on average, which validated the effectiveness of the aggregated census-derived HSAs.

**Fig. 7:**
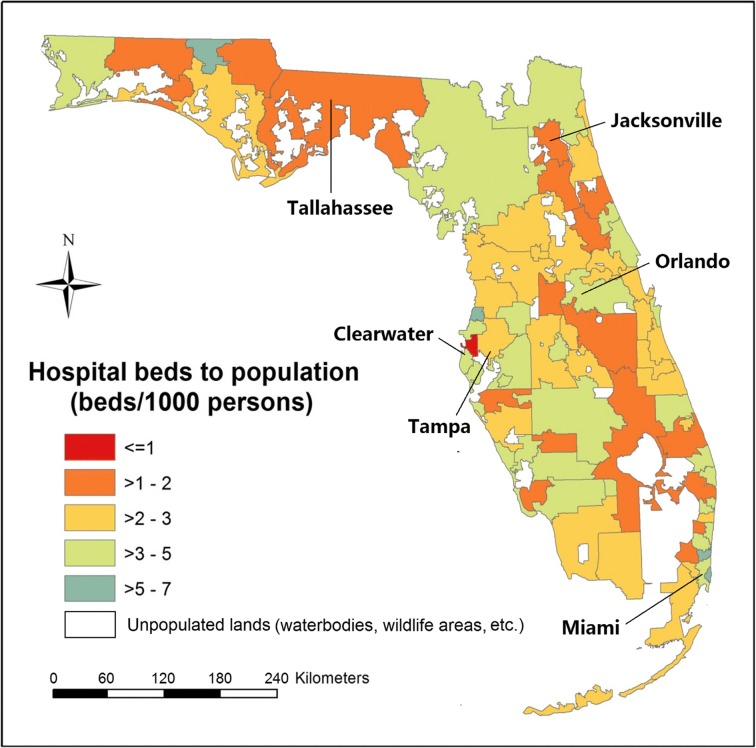
The ratios of hospital beds to population within the aggregated census-derived HSAs

The ratio difference between the most over- and under-served HSA units was about ten times, i.e., 7.2 in Graceville (to the west of Tallahassee) versus 0.7 in Safety Harbor (to the east of Clearwater). This has decreased by about six times when comparing with the ratio difference among census-derived HSAs. The range of ratios also decreased by about six times, i.e., ≤ 1 to 36 versus ≤ 1 to 36 (Fig. 7). Given no change in those significant clusters of HBtP ratios between two levels of HSAs (i.e., high in Miami and low in Jacksonville), it seems that more spatial heterogeneities were hidden at the aggregated level.

## Discussion

This study, from an angle of promoting the equity between hospital demand and supply, incorporated census data with hospital data for developing an approach to produce the census-derived HSAs in Florida as compared to that produced with only patient visit data. A large geographical disparity in hospital resource allocation was observed across HSAs. Also, a strong positive correlation between the numbers of hospital discharges and the population was found at ZIP code level. Despite being a by-product during the development of the HSA delineation approach, this finding is of equal importance to the spatial heterogeneity in per capita share of hospital capacity. Such a high correlation can substantially facilitate affordable population estimation, especially in non-census years. Although the 5-year population estimates at the CT and BG levels became available through the American Community Survey in 2005, they were still derived from Census population counts and thus remained limited in temporal resolution and accuracy (especially in less populated areas). Given an inherent relationship between patients and the population, the increasingly available patient data annually in the USA (e.g., SID, State Ambulatory Surgery and Services Databases, State Emergency Department Databases) could be potentially utilized to pursue the annual population estimates with higher accuracy.

The skeleton of this delineation method consists of iterative spatial intersection, computation, neighbor searching, and network analysis, which have been greatly supported and facilitated by GIS and could be easily automated. The census data and GIS have been used together to advance many aspects of population and health research (Jia et al. 2014, 2016; Jia and Gaughan 2016; Luo and Wang 2003); however, no efforts have been made to delineate or improve the HSAs for specific purposes, such as monitoring the equity of hospital resources between supply and demand (a surplus of beds, if not considering operation costs and physician and nurse workforce, could cause an overutilization of hospital services as stated in Roemer’s Law (Shain and Roemer 1959)).

This method has two major advantages over the previous approaches of HSA delineation (Center for Evaluative Clinical Sciences 1999; Jia et al. 2015, 2017a, b; Jia 2016; Klauss et al. 2005). First, previous approaches produce HSAs that usually contain multiple hospitals in one unit, which creates a higher level of difficulty in assigning responsibility for variation to individual hospitals due to lack of the HSA managers who can coordinate all hospitals within an HSA and manage the HSA as a whole. The census-derived approach seeks to form as many separate HSAs with as few hospitals within each HSA as possible, where each hospital could have its own HSA as long as it serves the highest percentage of patients in its ZIP code. This greatly reduces unnecessary complexities when focus is on the population-related demand for healthcare resources (e.g., hospital beds, physicians), which may interest relevant stakeholders and contribute to planning and optimization of hospital resource allocation and healthcare policy-making.

Second, previous approaches fail to consider unrecorded flows and potential demand within HSAs (Brown and Hincks 2008). More often than not, besides the largest percentage of discharges from a certain hospital to a given ZIP code, the percentage of the remaining discharges in that ZIP code from other hospitals may still be considerable. For example, imagine a ZIP code where 51% of discharges are from one hospital and 49% are from other(s). Previous approaches only consider the largest percentage and simply assign all discharges in that ZIP code to the hospital which discharges only 51% of patients, which would result in the biased estimation of patient numbers within HSAs. Such bias may propagate its ways into evaluation of local hospital resource allocation as multiple hospitals in one HSA could complicate assigning the responsibility to each hospital, resulting in inappropriate intervention and policy making. The census-derived approach enables BGs, used as ancillary data, to split up that ZIP code into multiple intersected zones and assigns each intersected zone to the nearest hospital (or adjacent HSA). Therefore, boundaries of the census-derived HSAs should be less subject to fluctuations over time than of HSAs based on patient travel flows, which might present only a transient reflection of unknown demand at a certain point in time.

Although a value of 50% has been used as a minimum cutoff percentage (of discharges) in previous delineation methods for assigning a ZIP code to a hospital (Jia et al. 2015; Jia 2016; Klauss et al. 2005), a one-size-fits-all cutoff value remains debatable. In this study, whether the largest percentage was twice the second largest percentage of discharges from a different hospital determined whether a ZIP code would be assigned to the hospital discharging the largest percentage of its patients. Such a relative and unconstrained cutoff percentage could further prevent bias caused by assigning a ZIP code with comparative percentages of discharges from different hospitals to only one hospital. This provides more flexibility at different locations than an absolute and one-size-fits-all percentage.

There are some limitations in this study. First, the population and discharges might not be homogeneously distributed within ZIP codes. Thus, there exists uncertainty in the estimated numbers of discharges within BGs. The correlation between the numbers of hospital discharges and the population also remains in doubt at other geographic levels and in other regions, which needs to be verified in more studies. Another major source of uncertainties is the subjective thresholds assigned when deciding whether a ZIP code should be assigned to one or multiple hospitals (i.e., 200%) and whether assigning should end (i.e., 10%). Second, in addition to the population number, other demographic information such as age and gender may affect the demand for healthcare supply and should be considered in future efforts. For example, elderly people are usually hospitalized more often and multiple times than adolescents. Also, the high numbers of “snowbird” population (temporary residents), veterans population, and both US and international tourists in Florida should be considered in future studies. Third, 13 unidentified hospitals and 47 psychiatric, rehabilitation, children’s, women’s, and other specialty hospitals (including about 5.1% of all discharging records) were excluded from this study, which might affect results. Besides the number of hospital beds, more attention should be paid to the type and range of health services provided by hospitals, as well as outpatient care. Fourth, the patterns of HBtP ratios in this study were different from the patterns among counties or other HSA boundaries. This is also commonly known as the modifiable areal unit problem (MAUP) that conclusions drawn at one geographic level may change when a different analysis unit is used (Openshaw 1984). The relationships among these patterns at different scales need to be discussed further in future studies. Additionally, patients do not always visit the nearest hospitals, which could be affected by the competition among hospitals and other underlying factors (e.g., health insurance, hospital rank/reputation, quality of care). Thus, there exists uncertainty of patients’ decision making in hospital selection within ZIP codes, which may be tested by questionnaire survey at the local scale. More in-depth analyses are also warranted to examine the factors that might affect the hospital selection by patients. Moreover, HSA boundaries could inform which hospitals nearby might have impacts on residents at any location, and hence, spatial accessibility of hospital resources could be explored using floating catchment area metrics at finer scales.

## Conclusions

Patients’ addresses within ZIP codes will remain unavailable due to confidentiality protection that may last for an unexpectedly long period. On the basis of multidisciplinary principles and both actual hospital data and potential population data, this study expands the science of HSA delineation by demonstrating an application of GIS in healthcare planning. The resulting census-derived HSAs allow policy-makers to easily identify the geographical disparity in per capita share of hospital resources and assign responsibility to specific hospitals and areas, providing an appropriate spatial scale for evaluating and improving current healthcare planning and delivery. The method is particularly applicable for public health professionals and health services researchers. What is more, the strong correlation between numbers of hospital discharges and the population within ZIP codes makes each a good indicator for the other. This discovery holds a remarkable potential for affordable population estimation, especially in non-census years, and deserves to be substantially explored by incorporating other supplementary data sources (e.g., outpatient visit data).

## Electronic supplementary material

ESM 1 (DOCX 26 kb)
