# Supplementary material for: Teaming up census and patient data to delineate fine-scale hospital service areas and identify geographic disparities in hospital accessibility
Source: Environ Monit Assess. 2019 Jun 28;191(Suppl 2):303. doi: 10.1007/s10661-019-7413-4 (PMC6598966; doi:10.1007/s10661-019-7413-4)
Supplement: Supplementary file 1 — (DOCX 26 kb) [file 10661_2019_7413_MOESM1_ESM.docx]

**Appendix 1**

The *Dartmouth-derived HSAs* in Florida were produced based on overall patient data in 2011, by a traditional flow-based method termed *Dartmouth-Swiss hybrid method*. Firstly, hospital discharge records were aggregated into the volume of discharges from each hospital to each ZIP code, or conversely, considered as patient-to-hospital travel flows. On the other hand, each hospital was assigned to a ZIP code within which it was located; such ZIP codes were also referred to as *hospital regions*. Next, each remaining ZIP code was assigned to the *hospital region* that most patients in that ZIP code visited, and the ZIP codes assigned to the same *hospital region* were grouped into an initial HSA. Then, each disconnected ZIP code was re-assigned to an adjacent initial HSA, to ensure the geographic contiguity of all ZIP codes in one HSA. Lastly, each initial HSA with more patients visiting another initial HSA was merged into that initial HSA, also referred to as *plurality rule* (Center for Evaluative Clinical Sciences 1999), to produce the final *Dartmouth-derived HSAs*.

**Appendix 2**

Compared to geometric centroids, the population-weighted centroids have proved to be more accurately representative of the location of the population in large units, such as block groups (BG) and census tracts (Luo and Wang 2003; Wang 2015). Under an assumption that all patients lived at the most populated locations, the population-weighted centroid of a BG was calculated as follows:

*x_p_* = __/__ (1)

*y_p_* = __/__ (2)

where *x_p_* and *y_p_* are the *x* and *y* coordinates of the weighted centroid of a given BG *p*; *x_i_* and *y_i_* are the *x* and *y* coordinates of geometric centroids of the *i*th census block within *p* (census blocks are nested within BGs); *p_i_* is the population of the *i*th census block within *p*; and *n_p_* is the total number of census blocks within *p*.

**Appendix 3**

Similar as Appendix 2, under an assumption that all patients lived at the most populated locations, the population-weighted centroid of a hospital service area (HSA) was calculated as follows:

*x_p_* = __/__ (1)

*y_p_* = __/__ (2)

where *x_p_* and *y_p_* are the *x* and *y* coordinates of the weighted centroid of a given HSA *p*; *x_i_* and *y_i_* are the *x* and *y* coordinates of geometric centroids of the *i*th ZIP code within *p* (ZIP codes are nested within HSAs); *p_i_* is the population of the *i*th ZIP code within *p*; and *n_p_* is the total number of ZIP codes within *p*.

**References**

Center for Evaluative Clinical Sciences (1999). The Dartmouth Atlas of Health Care in the United States. In J. E. Wennberg (Ed.). Chicago, Illinois.

Luo, W., & Wang, F. (2003). Measures of spatial accessibility to health care in a GIS environment: synthesis and a case study in the Chicago region. *Environment and Planning B, 30*(6), 865-884.

Wang, F. (2015). Quantitative Methods and Socioeconomic Applications in GIS. In (pp. 60-61, 78). Boca Raton, FL: Taylor & Francis.
